# Neuropathological associations between translational control and Alzheimer's disease

**DOI:** 10.1002/alz70855_097357

**Published:** 2025-12-23

**Authors:** Mariana Chauvet, Danielle Cozachenco Ferreira, Alinny Isaac, Sergio T. Ferreira, Mychael V. Lourenco

**Affiliations:** ^1^ Federal University of Rio de Janeiro, Rio de Janeiro, Rio de Janeiro, Brazil; ^2^ Federal University of Rio de Janeiro, Rio de Janeiro, Brazil; ^3^ Institute of Biophysics, Federal University of Rio de Janeiro, Rio de Janeiro, Brazil; ^4^ Federal University of Rio de Janeiro, Rio de Janeiro, RJ, Brazil; ^5^ D'Or Institute for Research and Education, Rio de Janeiro, RJ, Brazil; ^6^ Institute of Medical Biochemistry Leopoldo de Meis, Federal University of Rio de Janeiro, Rio De Janeiro, Rio de Janeiro, Brazil

## Abstract

**Background:**

Alzheimer's disease (AD) is the leading cause of dementia in elderly humans worldwide. Brain mRNA translation (protein synthesis) is essential for synaptic plasticity and cognition, and converging evidence indicates it is impaired in AD. Protein synthesis is composed of three major steps: initiation, elongation and termination. There are several factors involved in all three steps that are essential to ensure proper translation control. In particular, the key factors of the initiation and elongation steps are the eukaryotic initiation factor 2 alpha (eIF2a) and eukaryotic elongation factor 2 (eEF2), respectively, whose aberrant phosphorylation indicates translational repression. Importantly, failures in translational control are associated with several pathological conditions, including AD. Our group and others have shown that global protein synthesis is attenuated in AD neurons. However, the precise roles of eIF2a and eEF2 (and their phosphorylation status) in AD and other causes of dementia are not yet fully understood.

**Method:**

Phosphorylated and total eIF2a and eEF2 were measured by Western blotting in postmortem hippocampi and prefrontal cortices from healthy controls (HC) and AD patients of a Brazilian cohort.

**Result:**

We found that phospho‐eIF2a and phospho‐eEF2 levels are increased in individuals with higher CERAD amyloid score in the hippocampus and prefrontal cortex. Similarly, both phospho‐eIF2a and phospho‐eEF2 levels are increased in individuals with higher Braak stages in the prefrontal cortex.

**Conclusion:**

Together, these results suggest that these translational factors may be differently altered in relation to amyloid and tau pathology in AD.

